# Near-real-time MODIS-derived vegetation index data products and online services for CONUS based on NASA LANCE

**DOI:** 10.1038/s41597-022-01565-2

**Published:** 2022-08-04

**Authors:** Chen Zhang, Zhengwei Yang, Liping Di, Eugene G. Yu, Bei Zhang, Weiguo Han, Li Lin, Liying Guo

**Affiliations:** 1 Center for Spatial Information Science and Systems, George Mason University, Fairfax, VA 22030 USA; 2 Department of Geography and Geoinformation Science, George Mason University, Fairfax, VA 22030 USA; 3 Research and Development Division, U.S. Department of Agriculture National Agricultural Statistics Service, Washington, DC 20250 USA

**Keywords:** Agriculture, Geography

## Abstract

This paper describes a set of Near-Real-Time (NRT) Vegetation Index (VI) data products for the Conterminous United States (CONUS) based on Moderate Resolution Imaging Spectroradiometer (MODIS) data from Land, Atmosphere Near-real-time Capability for EOS (LANCE), an openly accessible NASA NRT Earth observation data repository. The data set offers a variety of commonly used VIs, including Normalized Difference Vegetation Index (NDVI), Vegetation Condition Index (VCI), Mean-referenced Vegetation Condition Index (MVCI), Ratio to Median Vegetation Condition Index (RMVCI), and Ratio to previous-year Vegetation Condition Index (RVCI). LANCE enables the NRT monitoring of U.S. cropland vegetation conditions within 24 hours of observation. With more than 20 years of observations, this continuous data set enables geospatial time series analysis and change detection in many research fields such as agricultural monitoring, natural resource conservation, environmental modeling, and Earth system science. The complete set of VI data products described in the paper is openly distributed via Web Map Service (WMS) and Web Coverage Service (WCS) as well as the VegScape web application (https://nassgeodata.gmu.edu/VegScape/).

## Abstract (summary)

**Table:** 

Measurement(s)	vegetation index
Technology Type(s)	remote sensing

## Background & Summary

The vegetation index (VI) is an effective indicator of vegetation photosynthetic activity and canopy structural variations in the study of geosciences and remote sensing^1^. A variety of VIs, including Normalized Difference Vegetation Index (NDVI)^2^, Enhanced Vegetation Index (EVI)^3,4^, Leaf Area Index (LAI)^5,6^, have been derived from Earth observation data captured by different sensors. As one of the most widely used remote sensing data, Moderate Resolution Imaging Spectroradiometer (MODIS) has been providing daily observation of global land surface from 2000 to the present. With the high spatial/temporal resolution and data continuity, the MODIS data products have been widely used in time series analysis and change detection studies^7^. Specifically, it is an ideal data source for deriving VI to measure crop conditions^8^. The vegetation condition information derived from the MODIS data can provide timely decision support to vegetation phenology monitoring^9^, crop progress mapping^10^, crop type classification^11,12^, crop yield estimation^13–16^, cropping patterns^17^, flood and drought detection^18,19^, and other applications in agriculture.

Created from the surface reflectance bands, the MODIS VI Product (MOD13/MYD13)^20^ provides the Level-3 global mapping of NDVI and EVI with spatial resolution of 250 m, 500 m, and 1 km (https://modis.gsfc.nasa.gov/data/dataprod/mod13.php). One limitation of MOD13/MYD13 is that these products are only generated at 16-day and monthly intervals, which cannot meet the timeliness requirement for many agricultural applications and decision-making, especially during disastrous events, such as storms and floods, wildfires, and droughts, occur. On the other hand, the standard, science-quality Terra/Aqua MODIS products are normally available after 20–48 hours of observation. This latency could also potentially affect the real-time observation of crop conditions. To reduce the latency of MODIS data, NASA has launched the Land, Atmosphere Near-real-time Capability for EOS (LANCE) program, which provides Level 0–3 near-real-time (NRT) Terra/Aqua MODIS data within less than 3 hours of observation. Based on the LANCE-MODIS, the daily 8-day rolling VI products at the spatial resolution of 250 m (MOD13Q4N) and 500 m (MOD13A4N) are also available. However, the current NASA MODIS VI products only have daily NDVI and EVI, which cannot reflect the vegetation condition within the historical time series. Therefore, an operational NRT VI data set with plentiful index types and temporal options to meet the timeliness needs of forecasting and monitoring crop conditions is desirable by the public.

This paper describes a set of NRT VI products and its associated online service for the Conterminous United States (CONUS) based on NASA’s LANCE-MODIS. Updated and published within 24 hours of observation, this data set aims to meet the timely needs for vegetation condition monitoring for U.S. cropland. Meanwhile, we have archived the complete volume of historical VI data back to 2000. This continuous data set with more than 20 years of vegetation condition observation would be suitable for time series analysis in many research fields such as agriculture, remote sensing, geographical information science and systems, environmental modeling, and Earth system sciences.

## Methods

### Workflow

**Fig. 1:**
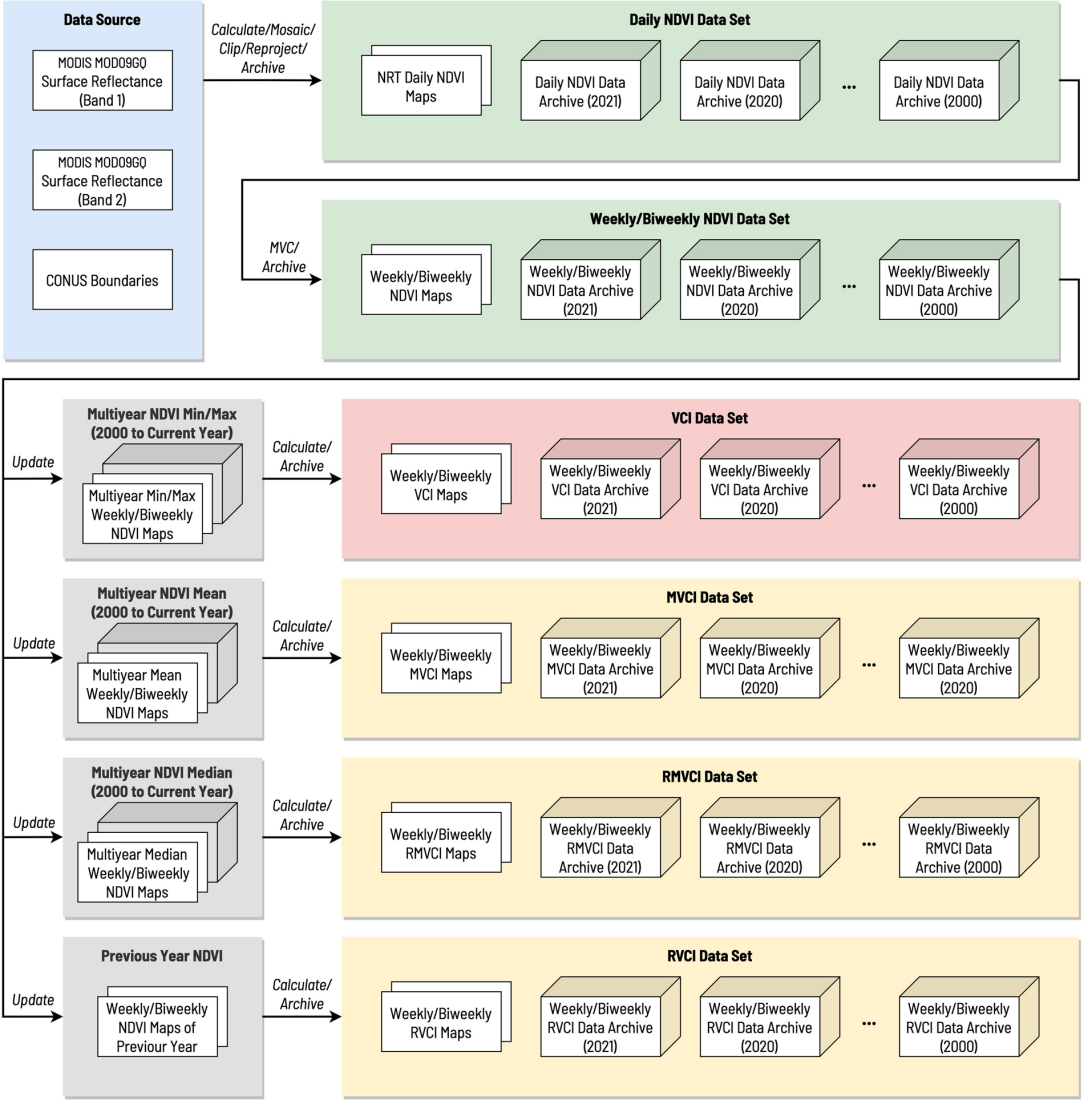
Overall workflow for the production of NDVI, VCI, MVCI, RMVCI, and RVCI data products based on LANCE-MODIS.

The data set described in this paper covers five types of indices, Normalized Difference Vegetation Index (NDVI), Vegetation Condition Index (VCI), Mean-referenced Vegetation Condition Index (MVCI), Ratio to Median Vegetation Condition Index (RMVCI), and Ratio to previous-year Vegetation Condition Index (RVCI). Figure 1 shows the overall workflow for the production of all VI data products described in this paper. Based on the temporal resolution, our VI products can be grouped into the daily product (NDVI) and the weekly/biweekly products (NDVI/VCI/MVCI/RMVCI/RVCI). The main source of the daily NDVI is the LANCE-MODIS data. Other VI products are derived from the daily NDVI data. The rest of this section will describe the details of calculation and processing for NDVI, VCI, and MVCI/RMVCI/VCI data products.

### NDVI

NDVI is the most widely used measurement to quantify vegetation conditions of remotely sensed imagery. The daily NDVI data described in this study are derived from the RED band and the NIR (near-infrared) band of MODIS data. It is noteworthy that the NRT daily NDVI data described in this study are produced based on the MODIS NRT Terra Surface Reflectance Daily Level-2G Global 250 m product (MOD09GQ NRT v061, dataset 10.5067/MODIS/MOD09GQ.NRT.061) within one day of observation. The archived daily NDVI from 2000 to 2019 and the derived weekly VI data are produced based on the MODIS Terra Surface Reflectance Daily Level-2G Global 250 m product (MOD09GQ v061, dataset 10.5067/MODIS/MOD09GQ.061). Table 1 summarizes the information on surface reflectance bands for the MOD09GQ NRT product.

**Table 1:** Surface reflectance bands for the MOD09GQ NRT product.

HDF Layer	Data Type	Valid Range	Wavelength	Spatial Resolution	Description
Surface reflectance band 1	16-bit signed integer	−100 to 16000	620 to 670 nm	250 m	RED band
Surface reflectance band 2	16-bit signed integer	−100 to 16000	841 to 876 nm	250 m	NIR band

The first step of NRT VI calculation is to mosaic the daily MODIS images and clip the area to the CONUS. Then the NDVI is calculated using surface reflectance bands of the MODIS data, which is defined as:

$$NDV{I}_{i}\left(x,y\right)=\frac{NI{R}_{i}\left(x,y\right)-Re{d}_{i}\left(x,y\right)}{NI{R}_{i}\left(x,y\right)+Re{d}_{i}\left(x,y\right)}$$

where *NIR*_*i*_ and *Red*_*i*_ represent the surface reflectance value for the near-infrared band and red band of each pixel. Based on the above equation, the NDVI value ranges from −1 to 1. The common NDVI value range for vegetation is 0.2 to 0.8^21^. The weekly and biweekly NDVI data are calculated by picking up the maximum daily raw NDVI in the 7-day/14-day period for every week/two-week using the maximum value composite (MVC) method. This is different from the rolling 8-day average method used in MODIS VI products (MOD13Q4N/MOD13A4N). The MVC method will keep the pixel of the maximum value from daily NDVI maps within a week or two weeks. This method can efficiently reduce the effect of bad pixels (e.g., cloud, fog, or no data) in a single daily NDVI.

To reduce the file size, we converted the NDVI data type from float to integer by scaling the index range from [−1, 1] to [0, 250], which can be defined as:

$$NDV{I}_{scaled}(x,y)=NDV{I}_{i}\left(x,y\right)\times 125+125$$

### VCI

VCI is a vegetation index derived from historical NDVI^22^. It indicates the percentage of the difference between the current NDVI value and historical minimum NDVI value with the NDVI dynamic range, which is defined as:

$$VC{I}_{i}(x,y)=\frac{NDV{I}_{i}\left(x,y\right)-NDV{I}_{min}\left(x,y\right)}{NDV{I}_{max}\left(x,y\right)-NDV{I}_{min}\left(x,y\right)}$$

where NDVI_i_(x,y) represents the current NDVI value for the given pixel, *NDVI*_*max*_(*x, y*) and *NDVI*_*min*_(*x, y*) represent the maximum and minimum value of the historical NDVI time series for the given pixel.

Similar to NDVI data, the index range of VCI is rescaled from [0, 1] to [0, 250], which can be defined as:

$$VC{I}_{scaled}(x,y)=VC{I}_{i}(x,y)\times 250$$

### MVCI/RMVCI/RVCI

MVCI and RMVCI are indices measuring the deviation of the current year’s NDVI value to the mean and median value of the historical NDVI from 2000 to the current year. The equations of MVCI and RMVCI are respectively defined as:

$$MVC{I}_{i}(x,y)=\frac{NDV{I}_{i}\left(x,y\right)-NDV{I}_{mean}\left(x,y\right)}{NDV{I}_{mean}\left(x,y\right)}$$

$$RMVC{I}_{i}(x,y)=\frac{NDV{I}_{i}\left(x,y\right)-NDV{I}_{median}\left(x,y\right)}{NDV{I}_{median}\left(x,y\right)}$$

where *NDVI*_*i*_ *(x,y)* represents the current NDVI value for the given pixel. *NDVI*_*mean*_ *(x,y)* and *NDVI*_*median*_ *(x,y)* represent the mean and median value of the historical NDVI within the same period for the given pixel.

RVCI, on the other hand, stands for the ratio of the current year’s NDVI to the previous years within the same periods. The equation is listed as:

$$RVC{I}_{i}(x,y)=\frac{NDV{I}_{i}\left(x,y\right)-NDV{I}_{i-1}\left(x,y\right)}{NDV{I}_{i-1}\left(x,y\right)}$$

where *NDVI*_*i*_ *(x, y)* represents the NDVI value of the current year and *NDVI*_*i-1*_ *(x, y)* represents the NDVI value of the previous year.

To reduce the file size and remove the anomaly pixels, we limited the absolute value of the ratio to 125% and scaled the index range to [0, 250]. This conversion can be defined as:

$$V{I}_{scaled}(x,y)=\left\{\begin{array}{cc}0 & if\;V{I}_{i}\left(x,y\right)\le -1.25\\ V{I}_{i}\left(x,y\right)\times 100+125 & if-1.25 < V{I}_{i}\left(x,y\right) < 1.25\\ 250 & if\;V{I}_{i}\left(x,y\right)\ge 1.25\end{array}\right.$$

## Data Records

The data set described in this paper includes the up-to-date NRT VI products and the historical VI data derived from the archived MODIS data since 2000. The complete data set is deposited at Center for Spatial Information Science and Systems of George Mason University. Our NRT VI products are grouped into daily, weekly, and bi-weekly products based on temporal period. The latest daily NDVI data is normally produced and published within 24 hours of observation. The weekly and biweekly VI data are produced once the daily NDVI data within the start date and end date of the week are completed. Each data set is registered and assigned the Digital Object Identifier (DOI) by the International Society of Agromatics and distributed under the Creative Commons Attribution License. The records of all types of data products are summarized in Table 2.

**Table 2:** Summary of MODIS-derived NRT VI products.

Product	Spatial Resolution	Index Range	Scaled Index Range	No Data Value	Data Volume	DOI
NDVI (Daily)	250 m	[−1, 1]	[0, 250]	255	365–366 files (~35GB)/year	10.55130/data.HYEZ6201^27^
NDVI (Weekly/Biweekly)	250 m	[−1, 1]	[0, 250]	255	52–53 files (~5GB)/year	10.55130/data.OWHP6479^28^
Multi-year NDVI (Min/Max/Mean/Median)	250 m	[−1, 1]	[0, 250]	255	53 files (~5GB)	10.55130/data.SXBO9729^29^
VCI (Weekly/Biweekly)	250 m	[0, 1]	[0, 250]	255	52–53 files (~7GB)/year	10.55130/data.XMAD9899^30^
MVCI (Weekly/Biweekly)	250 m	[−1.25, 1.25]	[0, 250]	255	52–53 files (~3.5GB)/year	10.55130/data.MZXO5125^31^
RMVCI (Weekly/Biweekly)	250 m	[−1.25, 1.25]	[0, 250]	255	52–53 files (~3.5GB)/year	10.55130/data.YDWF4243^32^
RVCI (Weekly/Biweekly)	250 m	[−1.25, 1.25]	[0, 250]	255	52–53 files (~4.0GB)/year	10.55130/data.CYIL3313^33^

The data type of all VI products is 8-bit unsigned integer. The index of each data product has been scaled to the range from 0 to 250. The value of no data pixels is 255. There are 365/366 data files for the daily NDVI product as well as 52/53 data files for each weekly/biweekly VI product in a normal year. The multi-year NDVI products (mean/median/min/max) are dynamically updated with the production of new weekly/biweekly data while their file numbers are fixed (53 data files of each).

To make the data set findable, accessible, interoperable, and reusable (FAIR), all VI data products are also available through standard geospatial web service interfaces, including Web Map Service (WMS) and Web Coverage Service (WCS). The WMS/WCS is implemented using MapServer, where the endpoint can be accessed at https://nassgeo.csiss.gmu.edu/cgi-bin/mapserv. For example, the metadata of WMS capabilities for daily NDVI data of 2021 can be requested through https://nassgeo.csiss.gmu.edu/cgi-bin/mapserv?MAP=/WMS/NDVI-DAILY_2021.map&SERVICE=WMS&VERSION=1.3.0&REQUEST=GetCapabilities. To access the specific data record, users may change the map name in the request example according to data type and year. The naming convention of the WMS/WCS map file for all data products described in this paper is summarized in Table 3.

**Table 3:** Naming convention of MODIS-derived VI data products for WMS and WCS. (The YEAR option is the year from 2000 to 2021. The DATE/STARTDATE/ENDDATE option is the date in format of YYYY.MM.DD. The WEEK option is a two-digit week number from 01 to 53. The METHOD option for multi-year NDVI data can be one of MIN/MAX/MEAN/MEDIAN).

Product	Map File	Layer Name
NDVI (Daily)	NDVI-DAILY_*YEAR* (e.g., NDVI-DAILY_2021)	NDVI-DAILY_*DATE* (e.g., NDVI-DAILY_2021.01.01)
NDVI (Weekly)	NDVI-WEEKLY_*YEAR* (e.g., NDVI-WEEKLY_2021)	NDVI-WEEKLY_*YEAR*_*WEEK*_*STARTDATE*_*ENDDATE* (e.g., NDVI-WEEKLY_2021_01_2021.01.04_2021.01.10)
NDVI (Biweekly)	NDVI-BIWEEKLY_*YEAR* (e.g., NDVI-BIWEEKLY_2021)	NDVI-BIWEEKLY_*YEAR*_*WEEK*_*STARTDATE*_*ENDDATE* (e.g., NDVI-BIWEEKLY_2021_01_2021.01.04_2021.01.17)
Multi-year NDVI (Weekly)	NDVI-MULTIYEAR-WEEKLY	NDVI-MULTIYEAR-WEEKLY_*WEEK*_*METHOD* (e.g., NDVI-MULTIYEAR-WEEKLY_01_MEAN)
Multi-year NDVI (Biweekly)	NDVI-MULTIYEAR-BIWEEKLY	NDVI-MULTIYEAR-BIWEEKLY_*WEEK*_*METHOD* (e.g., NDVI-MULTIYEAR-WEEKLY_01_MEAN)
VCI (Weekly)	VCI-WEEKLY_*YEAR* (e.g., VCI-WEEKLY_2021)	VCI-WEEKLY_*YEAR*_*WEEK*_*STARTDATE*_*ENDDATE* (e.g., VCI-WEEKLY_2021_01_2021.01.04_2021.01.10)
VCI (Biweekly)	VCI-BIWEEKLY_*YEAR* (e.g., VCI-BIWEEKLY_2021)	VCI-BIWEEKLY_*YEAR*_*WEEK*_*STARTDATE*_*ENDDATE* (e.g., VCI-BIWEEKLY_2021_01_2021.01.04_2021.01.17)
MVCI (Weekly)	MVCI-WEEKLY_*YEAR* (e.g., MVCI-WEEKLY_2021)	MVCI-WEEKLY_*YEAR*_*WEEK*_*STARTDATE*_*ENDDATE* (e.g., MVCI-WEEKLY_2021_01_2021.01.04_2021.01.10)
MVCI (Biweekly)	MVCI-BIWEEKLY_*YEAR* (e.g., MVCI-BIWEEKLY_2021)	MVCI-BIWEEKLY_*YEAR*_*WEEK*_*STARTDATE*_*ENDDATE* (e.g., MVCI-BIWEEKLY_2021_01_2021.01.04_2021.01.17)
RVCI (Weekly)	RNDVI-WEEKLY_*YEAR* (e.g., RNDVI-WEEKLY_2021)	RNDVI-WEEKLY_*YEAR*_*WEEK*_*STARTDATE*_*ENDDATE* (e.g., RNDVI-WEEKLY_2021_01_2021.01.04_2021.01.10)
RVCI (Biweekly)	RNDVI-BIWEEKLY_*YEAR* (e.g., RNDVI-BIWEEKLY_2021)	RNDVI-BIWEEKLY_*YEAR*_*WEEK*_*STARTDATE*_*ENDDATE* (e.g., RNDVI-BIWEEKLY_2021_01_2021.01.04_2021.01.17)
RMVCI (Weekly)	RMNDVI-WEEKLY_*YEAR* (e.g., RMNDVI-WEEKLY_2021)	RMNDVI-WEEKLY_*YEAR*_*WEEK*_*STARTDATE*_*ENDDATE* (e.g., RMNDVI-WEEKLY_2021_01_2021.01.04_2021.01.10)
RMVCI (Biweekly)	RMNDVI-BIWEEKLY_*YEAR* (e.g., RMNDVI-BIWEEKLY_2021)	RMNDVI-BIWEEKLY_*YEAR*_*WEEK*_*STARTDATE*_*ENDDATE* (e.g., RMNDVI-BIWEEKLY _2021_01_2021.01.04_2021.01.17)

## Technical Validation

**Fig. 2:**
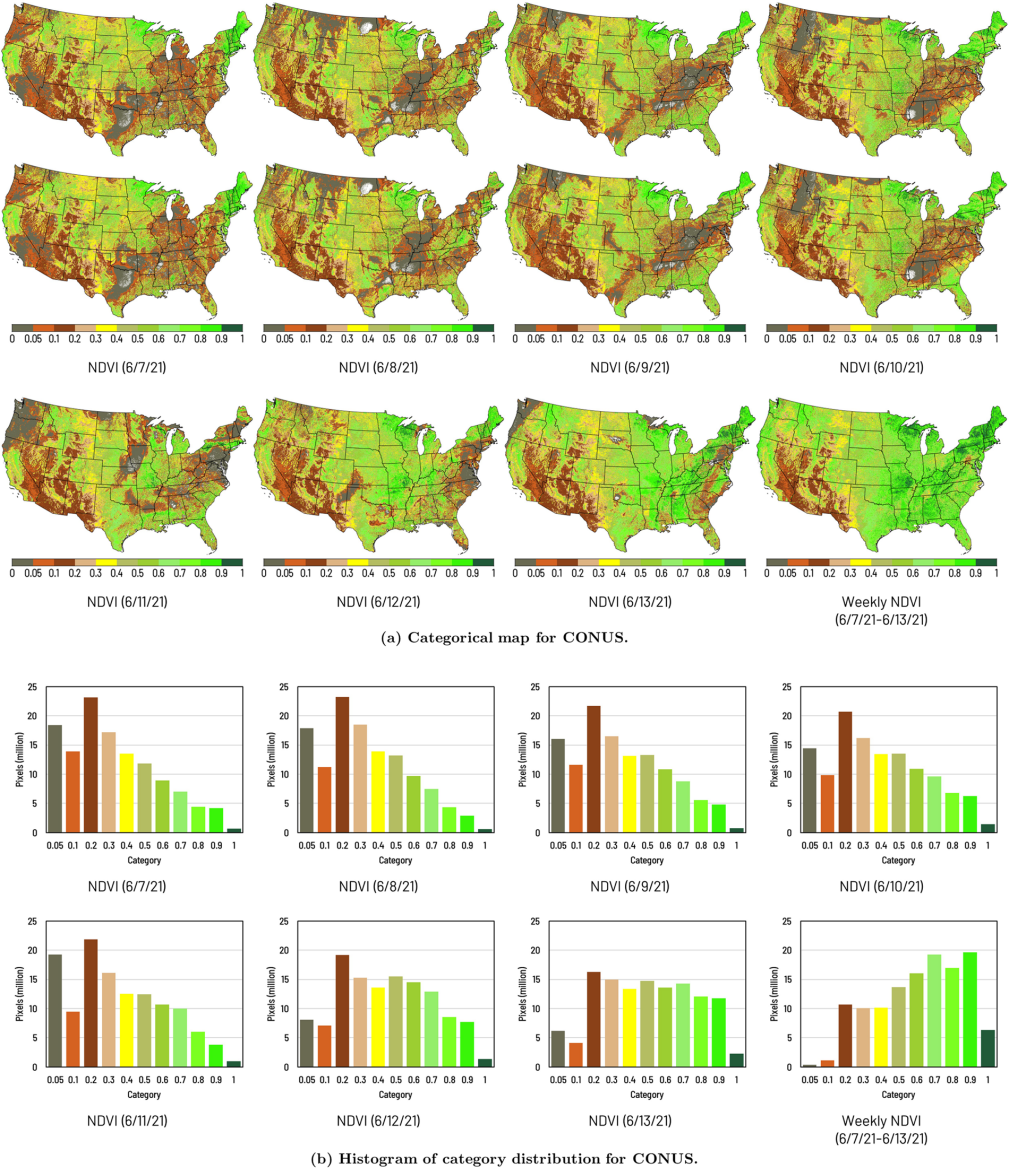
Example of daily and weekly NDVI data for CONUS. The index value of the NDVI categorical map ranges from 0 to 1 (values less than 0 are categorized as 0 to 0.05).

The sample datasets demonstrated in this data descriptor, including daily NDVI data from 6/7/2021 to 6/13/2021, and weekly/biweekly NDVI, VCI, MVCI, RMVCI, RVCI data for week 23 in 2021, are available on Figshare at 10.6084/m9.figshare.15130857.v5^23^. Figure 2 illustrates the validation of the NDVI data product. Figure 2a shows the categorical maps for daily NDVI data from 6/7/21 to 6/13/21 as well as the weekly NDVI data composited by daily NDVI data. The index value of the NDVI categorical map ranges from 0 to 1. Figure 2b shows histograms of category distribution for all daily and weekly maps. The mapping and quantitative results suggest the weekly NDVI map reflects the maximum value (the greenest pixels) in a week over the CONUS.

**Fig. 3:**
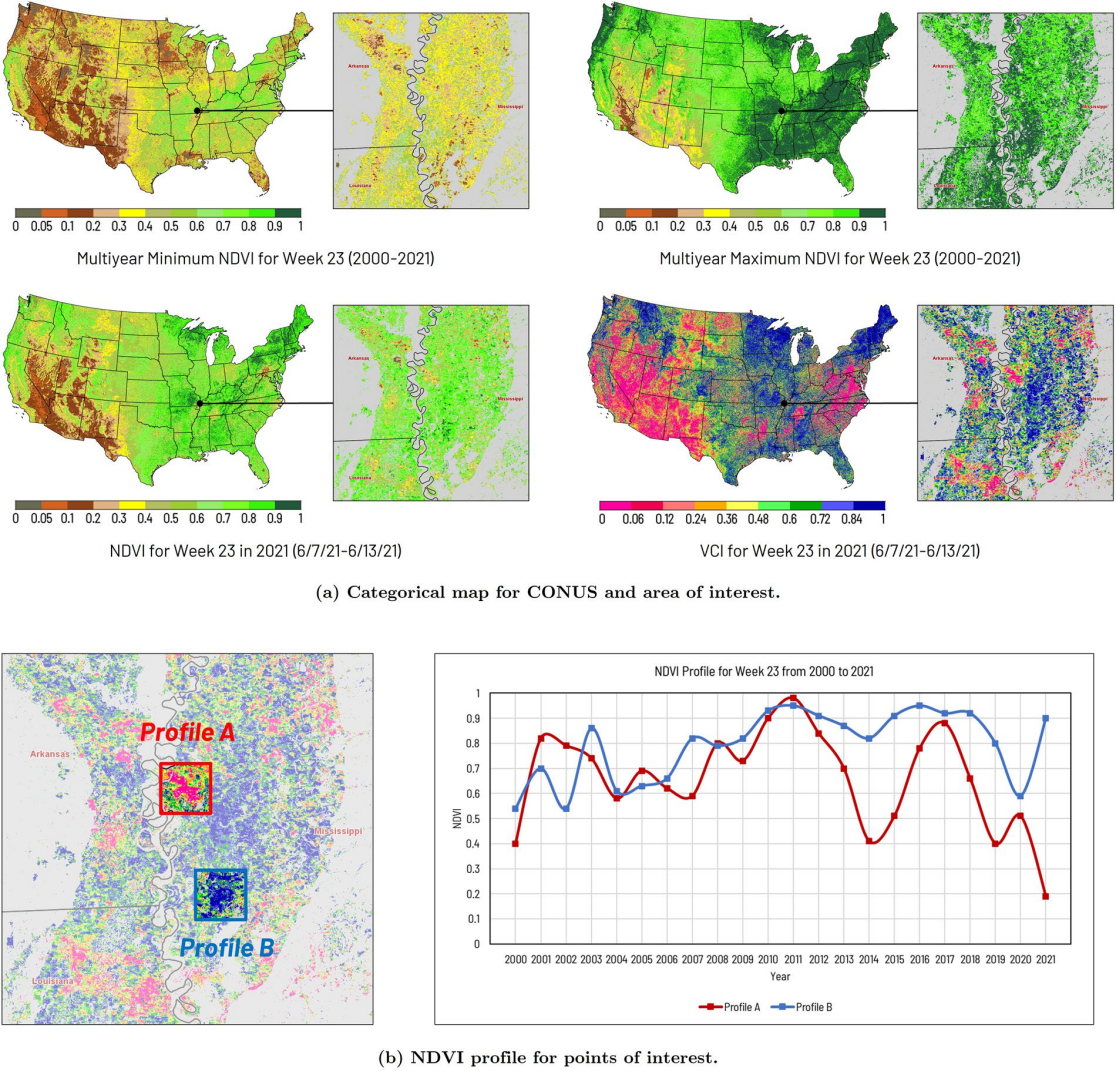
Example of weekly VCI data. The index value of the NDVI and VCI categorical map ranges from 0 to 1 (values less than 0 are categorized as 0 to 0.05). The cropland within the AOI is highlighted by the Crop Mask Layer derived from the 2017–2021 CDL data of USDA NASS.

The other weekly VI data products are calculated based on the weekly NDVI data products. As introduced in the method section, the VCI index is derived from the current week’s NDVI and the dynamic NDVI range for the same period in history. Figure 3 shows an example of VCI data derived from the NDVI data for week 23 in 2021 (6/7/21 to 6/13/21). The current week’s VCI, the current week’s NDVI, and the multi-year minimum/maximum NDVI composited from the historical NDVI for the same period are demonstrated in Fig. 3a. In the figure, the Delta Region, which mainly covers southeastern Arkansas, western Mississippi, and northeastern Louisiana, is selected as the area of interest (AOI) to demonstrate the detailed crop condition. In the detailed map, the cropland within the AOI is highlighted by the Crop Mask Layer derived from the 2017–2021 Cropland Data Layer (CDL) of USDA NASS^24^. The pink pixels suggest the cropland is suffering from severe vegetative drought stress, while the blue pixels indicate the cropland is in good vegetation state conditions. To further validate the VCI result, the NDVI profiles of two anomaly points are compared in Fig. 3b. Profile A indicates the vegetation condition of 2021 for the corresponding crop field is under the severe drought stress (NDVI < 0.2) in history. On the contrary, profile B shows the corresponding crop field had a good vegetation condition (NDVI > 0.9) in 2021.

**Fig. 4:**
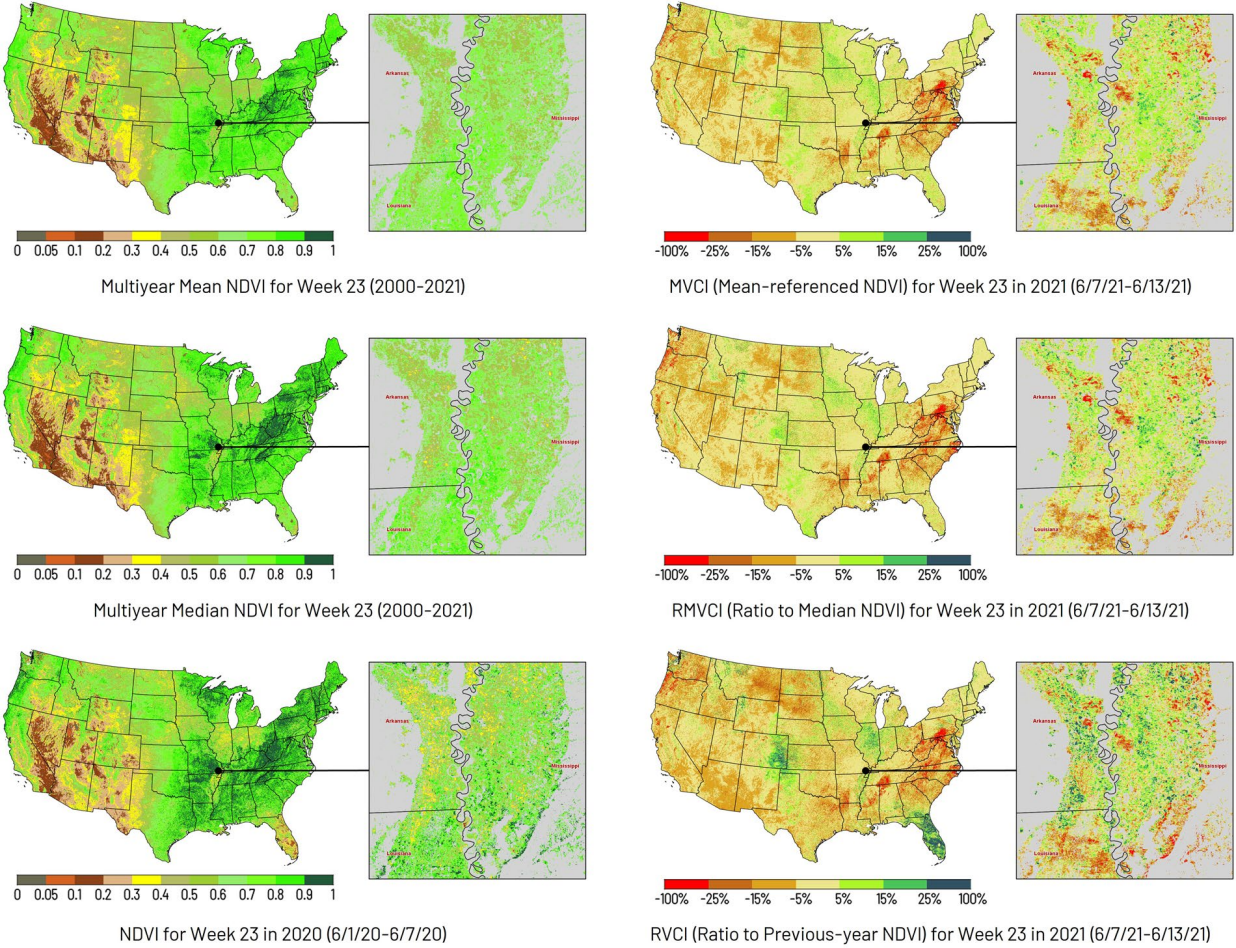
Example of weekly MVCI, RMVCI, and RVCI data. The index value of the NDVI categorical map ranges from 0 to 1 (values less than 0 are categorized as 0 to 0.05). The index value of the MVCI/RMVCI/RVCI categorical map ranges from −100% to 100% (values less than −100% are categorized as −100% to −25%, and values greater than 100% are categorized as 25% to 100%). The cropland within the AOI is highlighted by the Crop Mask Layer derived from the 2017–2021 CDL data of USDA NASS.

The MVCI, RMVCI, and RVCI are the ratios of the current week’s NDVI to the multi-year mean NDVI, multi-year median NDVI, and the previous year’s NDVI for the same period, respectively. Figure 4 compares the categorical map of MVCI, RMVCI, and RVCI with their referenced NDVI data. In the MVCI, RMVCI, and RVCI map, the red pixels suggest the cropland has a more severe vegetative drought stress condition than the referenced data. In contrast, the green pixels indicate the cropland has better vegetation conditions than the referenced data. Meanwhile, the absolute values of these indices indicate the anomaly of the current week NDVI.

## Usage Notes

**Fig. 5:**
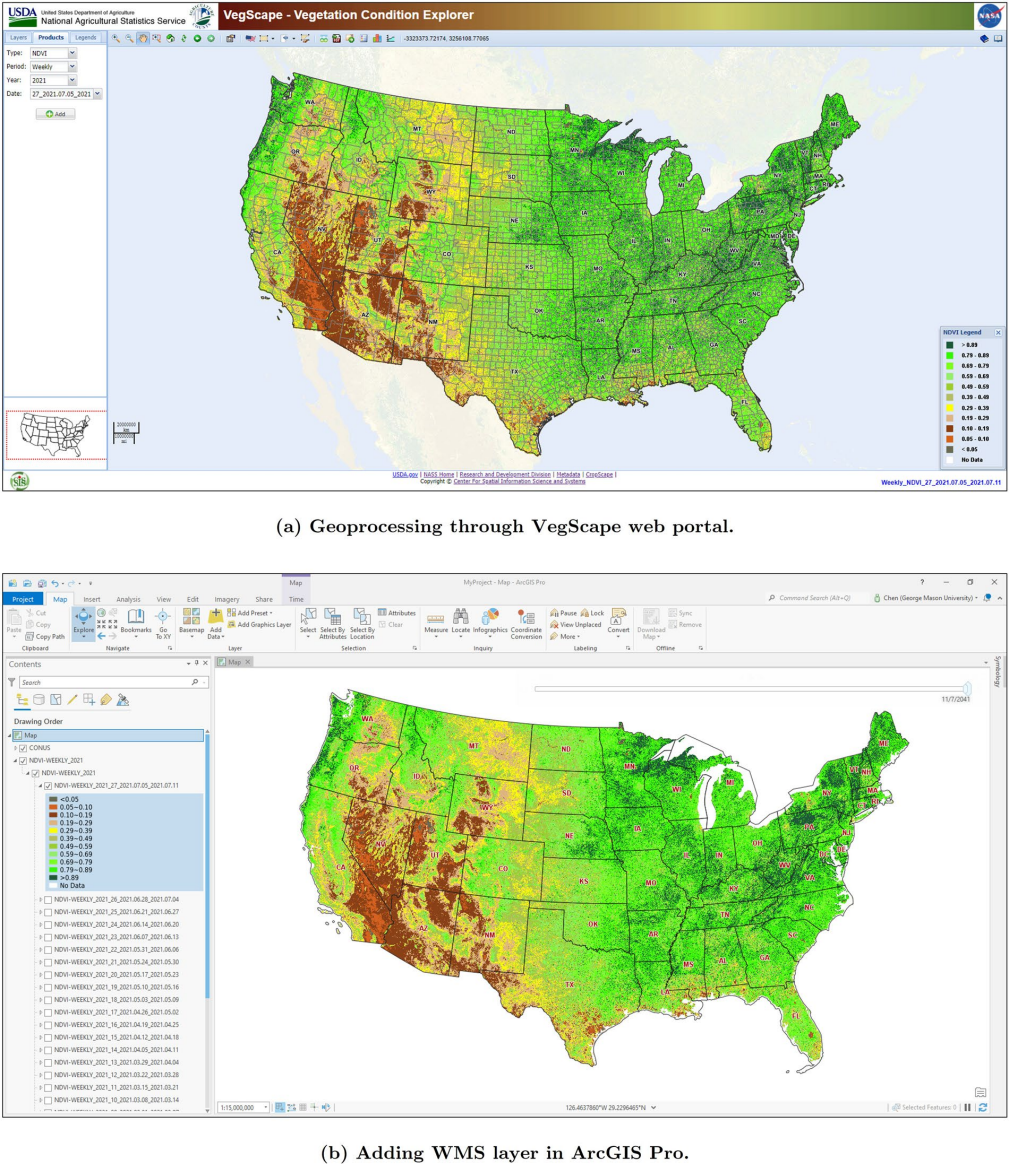
FAIR capability of VI data products with online service and desktop GIS software. The VegScape web application can be accessed at https://nassgeodata.gmu.edu/VegScape/. The GetCapabilities request of WMS layers demonstrated in ArcGIS Pro is available on https://nassgeo.csiss.gmu.edu/cgi-bin/mapserv?MAP=/WMS/NDVI-WEEKLY_2021.map&SERVICE=WMS&VERSION=1.3.0&REQUEST=GetCapabilities.

The VI data products described in this paper can be directly used in WMS/WCS compliant Geographic Information System (GIS) web applications and software. The complete VI data products are available on VegScape (https://nassgeodata.gmu.edu/VegScape/), a web-based online service operated and maintained by the Center for Spatial Information Science and Systems of George Mason University. This web client delivers a suite of FAIR geoprocessing and map services to explore and analyze U.S. crop condition^25,26^. In addition, all data records summarized in Table 3 are interoperable with desktop GIS software, such as ArcGIS and QGIS. Figure 5 demonstrates the FAIR capability of the VI data products through VegScape and ArcGIS Pro.

## Data Availability

Code, documentation, and other related sources used to produce VI data described in this work are available on GitHub at https://github.com/CSISS/NRT_VI. The usage and examples of requesting VI data through WMS/WCS are available on the Developer Guide page of VegScape at https://nassgeo.csiss.gmu.edu/VegScape/devhelp/help.html.
